# Supplementary material for: Patterns of Intron Gain and Loss in Fungi
Source: PLoS Biol. 2004 Nov 30;2(12):e422. doi: 10.1371/journal.pbio.0020422 (PMC532390; doi:10.1371/journal.pbio.0020422)
Supplement: Table S1 — Also available at http://genes.mit.edu/NielsenEtAl/. (4.3 MB ZIP). [file pbio.0020422.st001.zip › NielsenEtAl/html/697.html]

AN1536.1.NCU00499.1.MG04758.1.FG00515.1


```
 CLUSTAL W (1.82) Multiple Sequence Alignments - Introns Inserted


Sequence 1: NCU00499.1	629 aa
Sequence 2: FG00515.1	598 aa
Sequence 3: MG04758.1	639 aa
Sequence 4: AN1536.1	627 aa
Alignment Length: 680 aa
Number Identitical Residues: 139 aa
Alignment Score (without introns) 8346


MG04758.1 	MASTFQMP-VYYRPSALTVDTAHAQQKFFDDEDEGVLDDSIIDQS--AIDSGLDMSPALA
NCU00499.1	MGSATFQQPSYYRTPPLNVDTSCHGQKFFDDDE--ILDDDILNQS--TLNSALEMSPPLT
FG00515.1 	MSSTFQMQQPFYRPASLTLDT-QHAQKYFEDEDNSVLDDNVLDHN--TLDSGLELSPPMA
AN1536.1  	MARYSQPVFDFYHQHPSTLDTKTQPSAYPEDDEMSVLDDKILDTTSSDFTSPADHRRHSY
          	*.        :*:  . .:**    . : :*:: .:***.::: .:: : *  :      

MG04758.1 	DSRRESFAIG-SSLFSPKQED-WPSVDLQSLPTNNPFMSQQQNANPFMQAQHQSFMPQQQ
NCU00499.1	DSRRDSFAVSATALFSPKSDDSWPPVEMQSVPSNNPFFGQHNN-NPFMQMDQNQQSTYQN
FG00515.1 	DSRRDSFAVG-HPLFSPKTED-WQSVDMQSVPSNNPFLDQHSN-NPFMRLDQTQHNAYAT
AN1536.1  	EQGPDAFQHR-DSVWSDISQSLSSTQSRQNSQVGHPFFESAPN--PFMRMDGLPYVHSQQ
          	:.  ::*     .::*   :.   . . *.   .:**: .  *  ***: :         

MG04758.1 	NWVTMATSTPASTATAATFDGLP-VEFDSNPGSGFLRS----PIPFSNPTNNVAMFSSVN
NCU00499.1	-HWGM-MSSGAATP-LQSFDGLPQAEYESSTVPLFRPATVQAQTPFSNPNNQAVMFQQLG
FG00515.1 	ANWAFGPSSGACTP-LHPFDAMS-AEYDTN-VPLFQRP-MAGPTPFTNPGN---MFPALP
AN1536.1  	--WSISKDSGSCTP-SAMYENYP-TDMENTSVAPFAGGAVGPVNTVSMPSMTYRQHMAFA
          	    :. .: :.*.    ::  . .: :... . *   :     ..: *      .  . 

MG04758.1 	N--HSSSPASPQKDHWSGNMAMNK----KSRPDSPGIRSHNELRKGDGVRKKNARFDIPA
NCU00499.1	HHGIPAVPASPPKEHWINQELKNQAMAKRNRPTTPLIRSHNELRRGDGIRKKNARFDIPA
FG00515.1 	G-GNQSIPTSPQKEWMTPQQPINK---NKMRPGSPGIRSHNEMRRGDGIRKKNARFDIPA
AN1536.1  	PAGAVAMSPQSSQGWMPASTDMPDP-SSRPKNSPTYRNSSPLSVRRDGIRKKNARFEIPA
          	  .  : .... :     .    .. :.: :  ..  .*     : **:*******:***

MG04758.1 	ERNLANIDQLISQSTDEQEVKELKQQKRLLRNRQAA~LDSRQRKKQHTEQLEVEKKHYTN
NCU00499.1	ERNLSNIDQLIAQSTDEQEIKELKQQKRLLRNRQAA2LDSRQRKKQHTERLEDEKKHFTE
FG00515.1 	ERNLSNIDHLISQSTDEQEIKELKQQKRLLRNRQAA2LDSRQRKKQHTERLEDEKKQYTA
AN1536.1  	ERTLSNIDHLIAQSTNEEEIKELKQQKRLLRNRQAA2LDSRQRKKLHTEKLEEEKKHFTQ
          	**.*:***:**:***:*:*:**************** ******** ***:** ***::* 

MG04758.1 	VISNLEEELEDLRLTLQQMHTER-------SQMMARMQALEFDNEELVRRNIEETGELRR
NCU00499.1	VITNMEEQMNALQRDMDQLLREK-------QSYEAYISELKNEKEEIIREHTIETGELRK
FG00515.1 	VINDMEEEMAELRGKLEQMLLEK-------QTYTNYIENLNLEKEEMIRAHTIETGELRK
AN1536.1  	AINELEEELQNMRLREAELLREKEEWMATQQKISEYINSLHMDKDELIRAHTLETADLRK
          	.*.::**::  ::    ::  *:..  ::..     :. *. :::*::* :  **.:**:

MG04758.1 	QVKCLRTHIQDLEGSETSPSSGMSSMGAATSSFPGTFDGELDAINVEN-NWEHMGYMGDF
NCU00499.1	KVNVLTNHVQALESAAMSTPPAGPNV----SGFPGAYS-EMEGMAMDG-SWDNMPMFGEF
FG00515.1 	KVGVLTDHVQRLESAVPTATDN---------GFSSGFD-DMDGMGMPG-AWDASNFLNEY
AN1536.1  	KNNILKETVEKMERRVRTNVSN-------------EFS-DFENLTMESSPWEDFTMVNSL
          	:   *   :: :*    :                  :. ::: : : .: *:    ... 

MG04758.1 	QSASMDQAAVPEVKQEIVSRKPQTSQ----LVVSRRSDSTESEKPTTQGGVLFMLFLVGA
NCU00499.1	T----MEQQPSEVKQEMQIAPTKKSE----ISLS-----AENEKSPSQGGLLFMLFLVGA
FG00515.1 	P----AEP---EVKQEMAIATVKKSE----NPF------AESEK-PAQGGLLFMLFLVGA
AN1536.1  	SLDTDSVAPAAQPSQAMVVATNEKGSEKHAN------------EYPFSWNAFYMCLLFGA
          	   :      .: .* :     :..... :             : . . . ::* :*.**

MG04758.1 	LVCSSRS---MPSIPRVSEDVRAASETILHNVIKDAGIGEPTTSSFAMPSLPQASG-SLS
NCU00499.1	FVLSNRS---PPSIPRVSEDIRVASATLLDNVLKDAGLPQAASGLEAMA--PQPSGSSWA
FG00515.1 	FVLSSRS---TPAIPRVSEDVRVASANILDSVLKDAGVNSQSATLQAP---PQPSG---A
AN1536.1  	FIASNSASLPARSLPRLSEEYRAESANVLKAVLASSPPELAQSSSNQPP----VSSSVGL
          	:: *. :: .  ::**:**: *. * .:*. *: .:      :     .     *.:   

MG04758.1 	WPDSSAPGVSAQGLDGVVPSMLGDLSDFLTQPTSEQTKEHLFSMTAAQYDGMM-----AD
NCU00499.1	QSTGSSLPVAASGMNGVAPSMLGEMADALTQPTEQQTNEQLFGLTAAQYNGIS-----SQ
FG00515.1 	ANWGNPIAMNDMMMDGVAPSMLSELGDTLTQPTQEQTNEQIFSLSAAQYNGVN-----SQ
AN1536.1  	LPTTITGAEMAQMTGSAPTSNLDELHETLAMPTKEQEQEQAFALNAEQYNSLTTFDETGA
          	     .        ... .* *.:: : *: **.:* :*: *.:.* **:.: : ..:. 

MG04758.1 	GFLTSVPEDGERSTSQGRRNLAEALASATQS----NKAEVYTRSLLWDQIPRDVVKTFVK
NCU00499.1	DFLQNAPA--ERSTSKGRRNLAEALASMRAAANKDSAAEVYTRSLLWDKIPRDVVRDFAK
FG00515.1 	DFLQNAPE--KSSPSQGRRNLAEALAAMRSNKQSD-AADVYTRSLLWDQIPNDVVRNFAK
AN1536.1  	GYKSQQPSNLQQALAAMRGNAAQARTPLKAT------SDVYSRSLLWDRVPEKVIRDFRR
          	.:  . * . : : :  * * *:* :.          ::**:******::*..*:: * :

MG04758.1 	MVAECNQNACQPVGNDNDAHE
NCU00499.1	MVAESQQVAAA---NDADA--
FG00515.1 	MVSECNSAE------------
AN1536.1  	MVQEYGAPAARE---------
          	** *     .
```
